# Reductions in dental decay in 3-year old children in Greater Glasgow and Clyde: repeated population inspection studies over four years

**DOI:** 10.1186/1472-6831-11-29

**Published:** 2011-10-28

**Authors:** Alex D McMahon, Yvonne Blair, David R McCall, Lorna MD Macpherson

**Affiliations:** 1 Community Oral Health, Level 8, University of Glasgow Dental School, 378 Sauchiehall St, Glasgow, G2 3JZ, Scotland, UK; 2 NHS Greater Glasgow and Clyde, Oral Health Directorate, 120 Cornwall St South, Glasgow, G41 4AH, Scotland, UK

## Abstract

**Background:**

Dental decay remains one of the world's most prevalent diseases in childhood. It is unfortunate that the proportion of children suffering from oral disease is so high, given that dental decay is almost entirely preventable. The objective of this study was to examine dental inspection data from three-year old children to assess the extent to which the dental health in Greater Glasgow and Clyde had improved during the initial years of the Childsmile intervention programme.

**Methods:**

Dental inspections of three-year old children in Greater Glasgow and Clyde were undertaken in the academic years of 2006/7 and 2007/8 (the baseline years), and again in 2008/9 and 2009/10 (after the intervention had begun). A standardised protocol suitable for the age group was used. The number of decayed, missing and filled teeth was calculated (ie d_3_mft). If d_3_mft was > 0 then a child was said to have 'obvious decay experience' into the dentine. Additional results examined the effect of socioeconomic status using the Scottish Index of Multiple Deprivation (SIMD).

**Results:**

We inspected 10022 children (19% of the population). The weighted percentage of children with decay experience was 26% in 2006/7, 25% (2007/8), reducing to 18% (2007/8) and 17% (2009/10). When compared to the first baseline year of 2006/7, the OR was 0.91 for 2007/8 (0.79-1.06, p = 0.221), 0.63 for 2008/9 (0.55-0.72, p < 0.001), and 0.50 for 2009/10 (0.43-0.58, p < 0.001). The weighted mean d_3_mft was 1.1 in 2006/7, 1.0 in 2007/8 (p = 0.869), 0.6 in 2008/9 (p < 0.001) and 0.4 in 2009/10 (p < 0.001). Reductions in decay were seen in all socioeconomic groups.

**Conclusions:**

This study demonstrates that it is possible to impact upon the prevalence and morbidity of dental decay across the socioeconomic spectrum in a population. The dental health of young children in the Greater Glasgow and Clyde area has improved in recent years.

## Background

Dental decay remains one of the world's most prevalent diseases in childhood. The World Health Organisation estimates that dental decay affects between 60% and 90% of school age children and the vast majority of adults in developed countries [1]. Oral disease causes pain and much suffering for young children and often results in the necessity for general anaesthesia and surgical procedures. It is unfortunate that the proportion of children suffering from oral disease is so high, given that dental decay is almost entirely preventable. A previous editorial has noted that 'Preventing oral disease is important and achievable' and that 'preventive approaches exist, but they need to be rigorously promoted and implemented' [2]. Clearly, prevention of disease is a more important tactic than treating established disease [3]. This cliché is often used in a general sense in the field of medicine, but rarely is it so prescient. Regardless of political theory and tactics in implementing public health policy, it is of concern that so many young children have already experienced dental decay. This does not augur well for these children in later life. It has been shown that children with caries in their deciduous teeth have a greater risk of developing dental decay in their permanent teeth by the age of 12 [4].

Additional inspections of very young children at the start of their nursery education in NHS Greater Glasgow and Clyde have coincided with determined efforts by NHS Boards throughout Scotland to improve the dental health of all children. Two papers have recently been published which give a full account of the complex intervention known as the Childsmile programme [5,6]. The 'Demonstration' phase of the Childsmile programme covered the years 2006-2008. The current 'Interim' phase is intended to cover the years 2009-2011. The Childsmile programme consists of various interventions which are delivered at different stages of a child's life. The roll-out of the programme also proceeded in different sequences in different areas of Scotland.

Prior to the introduction of Childsmile a supervised nursery toothbrushing intervention with fluoride toothpaste was rolled out gradually from 1996 in NHS Greater Glasgow and Clyde. Over 90% of nursery establishments in the area were actively involved by 2006, and this coverage has continued to the present day [7]. By mid 2006 the Childsmile Practice component of the national programme commenced in this area and involved needs assessment of newborns by Public Health Nurses and referral of identified children at increased risk of future decay into Childsmile [5,6,8]. The programme involved a novel skill-mix i.e. Extended Duties Dental Nurses and community-based Dental Health Support Workers. These new staff enabled better integration of complementary interventions based in the community with those taking place in the dental practice setting. The aim was to facilitate provision of appropriate needs-based oral health support to infants and their families from the earliest age, either through facilitation to visit dental practice or via individually tailored home-support by the support workers, working as part of the extended Public Health nursing team. At the Childsmile dental practices the dental nurses provided and implemented individualised Oral Health Promotion care-plans for each child and family.

The Oral Health Promotion programme rolled-out in an incremental manner in the NHS Board area commencing earliest in the most deprived districts. The number of infants' first visits to dental practices increased five-fold between 2006 and 2009; in mid-2008 dental practices were offered a fee for providing fluoride varnish treatment to children enrolled in the programme, from their second birthday onwards; by the middle of 2009, 28000 children in deprived areas had been given Childsmile risk-assessments for future dental decay by their Public Health Nurses; 14000 children were enrolled into Childsmile dental practices or clinics, and 10000 had begun making visits to respective dental practices [6]. The objective of this study was to examine dental inspection data from three-year old children to assess the extent to which the 'basket' of activities comprising the Childsmile complex intervention had impacted on the dental health of three year old children in the NHS Greater Glasgow and Clyde area. The differential impacts across the spectrum of socioeconomic status were examined.

## Methods

Dental inspections were carried out in nursery schools in the first (known as the ante-pre-school year) of the two years of nursery education that are normally completed by Scotland's children. The NDIP inspections are usually carried out on five-year old children in Primary One and eleven-year old children in Primary Seven. Full NDIP reports from 2003 onwards are freely available online [9]. Additional NDIP dental inspections of three-year old children in NHS Greater Glasgow and Clyde have been undertaken in the academic years of 2006/7, 2007/8, 2008/9 and most recently in 2009/10. The NDIP data are generated as part of a Scottish Government oral health monitoring system and no further ethical approval is required for the analysis of this data. Legally, it is a statutory duty to inspect children at least twice. There is an option to opt-out of this programme but opt-in consent is not applicable.

A more detailed report of the methods and some results from 2006/7 and 2007/8 have previously been published [10]. In brief, the dental inspections were undertaken using standard British Association for the Study of Community Dentistry (BASCD) criteria, and all of the inspectors completed an annual training and calibration exercise immediately in advance of the dental inspections [11]. In the latest year of study we calibrated four examiners against an experienced 'gold standard' inspector, producing kappa scores for d_3_mft of 1.0, 1.0, 1.0, and 0.81 (based on one disagreement). The examiners had no knowledge of which components of the intervention had been experienced by individual children (or indeed in a school). After drying the teeth with cotton wool rolls and using standardised lighting conditions, the prevalence of 'obvious experience of dental decay which had extended into dentine' was recorded for each surface of each tooth. When using BASCD criteria, inspectors use a 'No. 4 plain' mirror and a CPITN probe (end diameter 0.5 mm). Probe use is restricted to removal of debris, detection of sealants and restorative materials and to determine cavitated lesions (greater than 0.5 mm in diameter). The data were assessed by calculating d_3_mft (number of decayed missing or filled teeth), d_3_t (number of teeth with unrestored decay extending into the dentine), mt (number of missing teeth, due to decay), and ft (number of teeth previously decayed, now filled). A Scottish Index of Multiple Deprivation (SIMD) score was ascribed to each child's data, using their home postcode. The SIMD (2006) contains 37 indicators of socioeconomic deprivation and has been recommended for all prospective analyses of Scottish health statistics [12,13]. The SIMD scores have been divided into quintile groupings i.e. SIMD 1 represents the most deprived areas and SIMD 5 represents the most affluent areas. Data for children with an unknown SIMD score were removed from the analysis cohort.

Population weights were calculated by the inverse of the probability of being sampled. The probability of being sampled is the number of children examined, divided by the equivalent number of children in the population in each of the five SIMD quintiles. This type of weighting is used to protect against inspection samples which might under-represent or over-represent one or more of the SIMD categories in the population. The mean d_3_mft and the percentage of children with no obvious decay experience in each SIMD quintile were analysed by survey methods, and the attendant 95% confidence intervals were calculated.

In addition, univariate and adjusted logistic models were used to calculate odds-ratios and 95% confidence intervals. In the adjusted models the effect of 'year of study' is adjusted by SIMD and age. In the univariate models, estimation of the c-index was used as a way of ranking the importance of the three variables in predicting dental decay, as all of the p-values were too small to be useful in this regard [14]. The c-index is the area under the curve of the lines of an ROC plot [15]. A variable with no predictive ability has a c-index of 0.5 and a variable with perfect predictive power has a c-index of 1.0. An interaction test between the effect of 'year of study' and SIMD was performed. Comparisons of the skewed d_3_mft data were carried out by Wilcoxon tests. Unadjusted mean d_3_mft statistics partitioned by SIMD categories for each year of study were also presented in a graph.

Bratthall has recommended using the 'Significant Caries Index' (SIC) to examine the distribution of d_3_mft in a population. The SIC score is the mean d_3_mft in the worst affected third of the population [16,17]. However, Morgan et al more recently recommend that a variety of cut-points should be used, with the choice depending on the research questions [18]. Thus, this study has used a variation of this method by calculating the mean d_3_mft for each twentieth of the population in ascending order of d_3_mft. A graphical presentation for each SIMD in each year of study was used to illustrate distribution of d_3_mft scores. The informative part of the graph is the tail of the distribution representing the children with the most dental decay. All analyses were carried out using SAS version 9.1 (SAS, Cary, NC), with survey analyses carried out using the SURVEYMEANS procedure.

## Results

The number of children with usable data was 1711 for 2006/7, 2428 for 2007/8, 3300 for 2008/9 and 2583 for 2009/10, giving a total of 10022 children for all of the years combined. Due to their young age some children refused to participate on the day of their inspections (9.6% on average). A total of 724 children were excluded due to the absence of a SIMD category. The breakdown by SIMD category of study subjects was 4688 with a score of 1 (the most deprived), 1669 with SIMD = 2, 1198 with SIMD = 3, 1004 with SIMD = 4, and 1463 children having the most affluent category of SIMD = 5. Thus the sample subjects were representative of the socioeconomic status of the background population [10]. Relative to the area's population of 3 year olds, the data analyses related to 14% of the population in 2006/7, 19% in 2007/8, 23% in 2008/9, and 19% in 2009/10.

The weighted results are presented in Table 1 for the percentage of children with obvious signs of dental decay experience, and in Table 2 for the mean d_3_mft (i.e. number of decayed, missing and filled teeth). The weighted percentage of children with decay experience in 2006/7 and in 2007/8 was 26% and 25%, respectively. This reduced to 18% in 2007/8 and 17% in 2009/10. In univariate analyses, the ranking of the usefulness of each variable was: SIMD c-index = 0.60, Age c-index = 0.56, and Year c-index = 0.55 (the children were all approximately three years old, which lowers the predictive ability of age in this study). The adjusted odds-ratio for decay experience in the most deprived areas versus the least deprived areas was 3.63 (95% confidence interval (CI) 3.03, 4.36, p < 0.001), and the adjusted odds-ratio for a 12 months increase in age was 2.27 (95% CI 1.92, 2.70, p < 0.001). When compared to the baseline year of 2006/7 the odds-ratios for prevalence of decay experience in subsequent years were 0.91 for 2007/8 (95% CI 0.79, 1.06, p = 0.221), 0.63 for 2008/9 (95% CI 0.55, 0.72, p < 0.001), and 0.50 for 2009/10 (95% CI 0.43, 0.58, p < 0.001). After adjustment for deprivation and age, a large reduction in decay experience over time was observed, with the odds of decay experience (i.e. d_3_mft>0) halving by the most recent year of study. Similar patterns in the results are evident from the analyses of mean d_3_mft (Table 2). The weighted mean d_3_mft was 1.1 in 2006/7, 1.0 in 2007/8 (p = 0.869), 0.6 in 2008/9 (p < 0.001) and 0.4 in 2009/10 (p < 0.001).

**Table 1:** % Obvious Dental Decay in Greater Glasgow and Clyde, Scotland

Year	SIMD*						
	1	2	3	4	5	Total	Weighted 95% CI
**2006/7**	216/655 (33%)	81/259 (31%)	56/247 (23%)	42/194 (22%)	46/356 (13%)	441/1711 (26%)	26%: 24%, 28%
**2007/8**	343/1068 (32%)	114/414 (28%)	68/296 (23%)	35/237 (15%)	65/413 (16%)	625/2428 (26%)	25%: 23%, 27%
**2008/9**	415/1581 (26%)	117/541 (22%)	42/333 (13%)	44/323 (14%)	34/522 (7%)	652/3300 (20%)	18%: 17%, 19%
**2009/10**	333/1384 (24%)	70/455 (15%)	46/322 (14%)	25/250 (10%)	9/172 (5%)	483/2583 (19%)	17%: 15%, 18%

Abbreviations: CI, confidence interval.

* SIMD = Scottish index of multiple deprivation, 1 = most deprived, 5 = least deprived.

**Table 2:** Mean d_3_mft (number of decayed, missing or filled teeth) in Greater Glasgow and Clyde, Scotland

Year		mean (standard deviation) for each SIMD*					
	1	2	3	4	5	Total	Weighted 95% CI
**2006/7**	1.5 (3.0)	1.3 (2.7)	0.9 (2.2)	0.7 (1.8)	0.3 (1.2)	1.1 (2.5)	1.1: 1.0, 1.2
**2007/8**	1.4 (2.9)	0.9 (2.0)	0.9 (2.2)	0.5 (1.6)	0.5 (1.5)	1.0 (2.4)	1.0: 0.9, 1.1
**2008/9**	1.0 (2.3)	0.7 (1.9)	0.4 (1.4)	0.4 (1.3)	0.2 (0.9)	0.7 (1.9)	0.6: 0.6, 0.7
**2009/10**	0.5 (1.2)	0.4 (1.1)	0.3 (0.9)	0.2 (0.8)	0.1 (0.4)	0.4 (1.1)	0.4: 0.3, 0.4

Abbreviations: CI, confidence interval.

* SIMD = Scottish index of multiple deprivation, 1 = most deprived, 5 = least deprived

**Figure 1:**
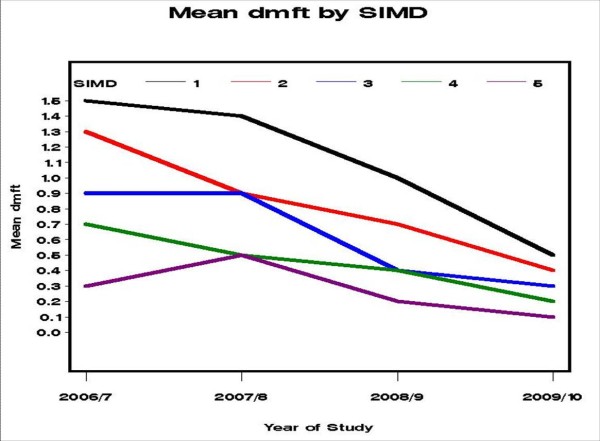
**Analysis of mean d_3_mft by Scottish Index of Multiple Deprivation (SIMD, 1 = most deprived, 5 = least deprived) in Greater Glasgow and Clyde, Scotland**.

**Figure 2:**
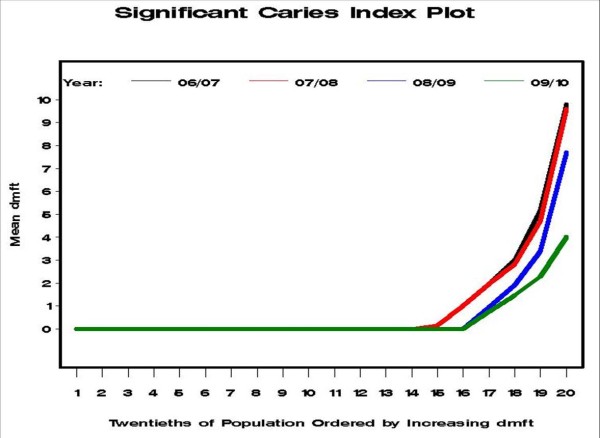
**Analysis of the Significant Caries Index, showing the mean dmft in each of the twentieth worst affected children in Greater Glasgow and Clyde, Scotland**.

Reductions in prevalence of dental decay experience were found in each of the five SIMD socioeconomic grades. For example, in the most deprived districts the percentage of children with obvious decay experience reduced from 33% (2006/7) to 24% (2009/10). The respective difference for children in the most affluent districts in the same time period was a reduction from 13% to 5%. There was a significant interaction between SIMD and year of study (p = 0.033), but this seems to have been generated by small differences in the odds-ratios being emphasised by the very large sample size. The mean d_3_mft reduced from 1.5 to 0.5 for the most deprived areas, and reduced from 0.3 to 0.1 in the most affluent areas. The trends for each SIMD category can also be seen graphically in Figure 1. The mean d_3_mft in each of the twentieths of the distribution of d_3_mft is given for each year in Figure 2. It can be seen that the curves for the twentieths with a mean of zero have moved towards the right, and the height of the tail has reduced progressively for the most recent two years. The actual mean d_3_mft in the 'worst twentieth', at the right of the plot, was 9.8 for 2006/7, 9.6 for 2007/8, 7.7 for 2008/9, and 4.02 for 2009/10.

## Discussion

This study has examined patterns of dental decay in four consecutive academic years for three-year old children in the NHS Greater Glasgow and Clyde area in the West of Scotland. The combined sample size of more than 10000 children, and the magnitude of the sampling fraction of the population, is large enough to make solid inferences and to create sub-groups using an area-based measure of socioeconomic status, such as the Scottish Index of Multiple Deprivation (SIMD). This is not a small research project that has tested whether a treatment may prove beneficial for patients with a particular diagnosis. By use of a 'directed-population' approach with differentially intensive interventions across the socioeconomic spectrum the Childsmile programme aims to promote measurable improvements in dental health at the level of the entire population [8,19-21]. In total, 10022 children (with deprivation data) were inspected, equivalent to an average of 19% of the entire eligible population of three year old children. The weighted proportion of children with obvious decay experience improved progressively over the two most recent years, so that ultimately decay experience reduced from 26% in 2006/7 to 17% in 2009/10. In a logistic regression model which adjusted directly by SIMD and age, the relative reduction appeared to be even greater, namely 50% which corresponds to the odds-ratio (OR) of 0.5 (note that the unadjusted OR = 0.66, adjusted by age OR = 0.61, adjusted by SIMD OR = 0.55, adjusted by both age and SIMD OR = 0.50). In other words, the relative reduction in the children studied is estimated at 50%, and in the population at large (weighted by SIMD but unadjusted by age) it is estimated at 35%. Presumably, if the entire population had been inspected the true population reduction would lie between these two estimates.

The relative improvements in dental health were seen across the socioeconomic spectrum, which is precisely what the recent independent reports for the World Health Organisation and the UK government advocate if health inequalities are to decrease in the future [22,23]. It can be seen in Figure 2 that reductions were clearly evident in even the worst affected twentieth of the distribution, as measured by d_3_mft. This is arguably because Childsmile adopts a life-course approach commencing at the earliest age with differentially intensive interventions customised to meet individuals' assessed oral health needs [23]. The Childsmile interventions tailored for individuals are over and above the universal programme elements e.g. daily supervised 1000 ppm F toothbrushing in all nurseries. The absolute improvement in obvious decay experience was 9% for the poorest areas and 8% for the most affluent areas (Table 1). In considering mean d_3_mft scores, it appears that the poorest children have benefited the most with an absolute improvement of 1.0 compared to only 0.2 for children in the most affluent areas (Table 1, Figure 1).

It is interesting that the weighted reduction in mean d_3_mft in the overall population is more noticeable between 2008/9 and 2009/10 (0.7 and 0.4, respectively) than that for the proportion with obvious decay experience (18% to 17%). Generally, we expect a strong relationship in dental health between mean d_3_mft and the percentage of subjects with decay [24]. However, a short lag is plausible in a backdrop of decreasing dental decay as this could follow Sheiham's Rule that 'as caries prevalence falls, the less susceptible sites (proximal and smooth surfaces) reduce by the greatest proportion, whilst the most susceptible sites (occlusal) reduce by the smallest proportion' [25]. In other words, some teeth and tooth surfaces are more resistant to improvement in a population than others, which militates against children becoming completely free of caries. However, in the directly adjusted logistic model the odds-ratio for 2008/9 versus 2006/7 was 0.63, and that for 2009/10 was 0.50, so there is some encouraging evidence of a progressive drop in obvious decay experience. Furthermore, there are sound theoretical reasons for the above magnitudes of improvement to have happened in NHS Greater Glasgow and Clyde following such interventions [26]. It is anticipated that these improvements will continue once Childsmile is embedded into the NHS Scotland remuneration system.

These results contrast sharply with those of a recent paper 'Giving Children a Healthy Start' by the Audit Commission [27]. It is of concern that the Audit Commission reports that in spite of a £7.2 billion investment in England's Sure Start (1998/99-2010/11), five-year-olds' mean d_3_mft values are deteriorating both in England as a whole and in their specially targeted 'Spearhead areas' (+0.29 d_3_mft, i.e.+19.9%). These trends contrast markedly with those observed for NHS Greater Glasgow and Clyde over the same period and are suggestive that the trends described here have not been part of a wider secular trend. Moreover, it has been considered that there is a pending wider public health crisis in many countries due to contemporary increases in children's dental caries prevalence [28].

## Conclusions

This study demonstrates that it is possible to impact upon both the prevalence and morbidity of dental decay across the socioeconomic spectrum in a population. Surely this type of 'anticipatory care' model is an improvement, when compared to the traditional model of 'reactive dental care' and is worthy of consideration for implementation elsewhere [3]. In the past, West of Scotland children from deprived backgrounds first experience of dental services was frequently for 'crisis care' [29]. Furthermore, a recent independent review noted that 'the NHS in 2009 is still dealing with, and paying for, the consequences of disease that developed more than 50 years ago' and making a transition in tactics will be a challenge [30]. At present, NHS Greater Glasgow and Clyde is the only NHS Board in Scotland to conduct epidemiological studies of its resident three-year-olds. This endeavour has helped produce our conclusion that the dental health of three year old children in the NHS Greater Glasgow and Clyde area has improved in recent years.

## Competing interests

The authors declare that they have no competing interests.

## Authors' contributions

All authors contributed to the design of the study and interpretation of the data. ADM wrote the first draft and conducted the statistical analyses. YB trained and calibrated the dental inspectors. All authors contributed to the writing of the manuscript, and have read and approved the final manuscript.

## Pre-publication history

The pre-publication history for this paper can be accessed here:

http://www.biomedcentral.com/1472-6831/11/29/prepub
